# Fruit Characteristics of Citrus Trees Grown under Different Soil Cu Levels

**DOI:** 10.3390/plants11212943

**Published:** 2022-11-01

**Authors:** Xiaorong Mo, Chuanwu Chen, Muhammad Riaz, Mohamed G. Moussa, Xiangling Chen, Songwei Wu, Qiling Tan, Xuecheng Sun, Xiaohu Zhao, Libiao Shi, Chengxiao Hu

**Affiliations:** 1 Key Laboratory of Arable Land Conservation (Middle and Lower Reaches of Yangtze River), Ministry of Agriculture, Huazhong Agricultural University, Wuhan 430070, China; 2 College of Resources and Environment, Beibu Gulf University, Qinzhou 535011, China; 3 Guangxi Laboratory of Germplasm Innovation and Utilization of Specialty Commercial Crops in North Guangxi, Guangxi Academy of Specialty Crops, Guilin 541004, China; 4 College of Resources and Environment, Zhongkai University of Agriculture and Engineering, Guangzhou 510225, China; 5 Soil and Water Research Department, Nuclear Research Center, Egyptian Atomic Energy Authority, Cairo 13759, Egypt; 6 Horticulture Research Institute, Guangxi Academy of Agricultural Sciences, Nanning 530007, China

**Keywords:** citrus, copper, pericarp color, sugar, acid

## Abstract

The effects of the increased soil copper (Cu) on fruit quality due to the overuse of Cu agents have been a hot social issue. Seven representative citrus orchards in Guangxi province, China, were investigated to explore the fruit quality characteristics under different soil Cu levels and the relationship between soil-tree Cu and fruit quality. These results showed that pericarp color a value, titratable acid (TA), and vitamin C (Vc) were higher by 90.0, 166.6, and 22.4% in high Cu orchards and by 50.5, 204.2, and 55.3% in excess Cu orchards, compared with optimum Cu orchards. However, the ratio of total soluble solids (TSS)/TA was lower by 68.7% in high Cu orchards and by 61.6% in excess Cu orchards. With the increase of soil Cu concentrations, pericarp color a value and Vc were improved, TA with a trend of rising first then falling, and TSS/TA with a trend of falling first then rising were recorded. As fruit Cu increased, pericarp color a value and TSS reduced and as leaf Cu increased, TSS/TA decreased while Vc was improved. Moreover, a rise in soil Cu enhanced leaf Cu accumulation, and a rise in leaf Cu improved fruit Cu accumulation. Fruit Cu accumulation reduced fruit quality by direct effects, leaf Cu improved fruit quality by direct and indirect effects. Soil Cu affected fruit quality by indirect effects by regulating leaf Cu and fruit Cu. Therefore, reasonable regulation and control of soil Cu concentrations can effectively increase pericarp color, sugar, and acid accumulation in citrus fruit.

## 1. Introduction

Citrus is considered the largest fruit crop, which is widely grown in more than 140 countries around the world, with over 4000 years of cultivation history [1]. China is the most important citrus planting region in the world, with a citrus planting area of 2.4867 million ha and a yield of 41.3814 million tons, ranking first in 2018 [2]. Moreover, citrus is grown widely in Guangxi province, China, with the highest amounts of fruit yield (8.3649 million tons) and the biggest scale of planting area (0.388 million ha) in 2018 [3].

Currently, copper (Cu) pesticides have been widely used to control citrus canker, blackspot, termite brown spot, and fruit drop after flowering [4]. However, only a small amount of the sprayed Cu fungicides could be absorbed by trees, while the remaining fungicides are washed and dropped into the soil by rain [5,6], precipitated in the soil [7], and finally result in Cu accumulation in citrus orchards soil year after year [8,9]. For instance, soil available Cu concentrations of a Brazilian cocoa plantation has reached 600 mg kg^−1^ after 16 years of application of Cu fungicides [10]. Furthermore, total Cu concentrations can rise to 250, 1280, and 1500 mg kg^−1^ in vineyards of Florida (USA), France, and England, respectively [11,12], which are very high compared to the background value (10 mg kg^−1^). Likewise, the excess ratios of soil available Cu in some citrus orchards of Hubei, Jiangxi, Hunan, Zhejiang, and Guangxi provinces in China are up to 75.0% [13], 85.3% [14], 88.90% [15], 95.1% [14], and 100% [16], respectively. These results highlight that excessive Cu accumulation in fruit orchards is a common problem in China and abroad.

Cu is an essential micronutrient for plant growth and development [17,18], which plays a significant role in plant photosynthesis, electron transfer, lignin synthesis, cell wall metabolism, and environmental stress response [19,20]. Nevertheless, Cu is also a toxic heavy metal that inhibits plant photosynthesis, oxidizes proteins and nucleic acids, disrupts nutrient absorption, and interferes with fruit quality formation [21,22,23,24,25]. Generally, Cu toxicity in citrus is manifested by fibrous root rot and death, poor growth, and iron chlorosis in leaves [26,27]. Citrus fruit quality is negatively affected by Cu [28]. For instance, sweet orange leaves exhibit toxic symptoms after spraying cupric nitrate, resulting in a loss of fruit yield [29], which is associated with a drop in fruit numbers [26]. Moreover, the fruit Cu concentration of ‘Majiayou’ pomelo (*Citrus grandis* Osbeck) is negatively correlated with fruit weight and peel thickness [30]. Likewise, the leaf Cu concentrations of Satsuma mandarin (*Citrus unshiu)* and Jin Cheng lines (*Citrus sinensis* Osbeck cv. Jin Cheng) are negatively correlated with vitamin C (Vc) concentration in fruit [31].

At present, more attention has been paid to the effect of Cu on citrus growth, such as root morphology [24], leaf photosynthesis [32], tree mineral nutrients absorption [33], and plant Cu tolerance [34]. However, little information is available concerning the effects on fruit quality attributes under different soil Cu levels and how Cu affects fruit quality. In this study, seven representative citrus orchards in Guangxi province were selected to study (1) the characteristics of fruit quality in citrus under different soil Cu levels; (2) the effect of leaf Cu and fruit Cu accumulation on fruit quality; (3) relationship between soil Cu and tree Cu. Taking these results into account, we can more scientifically evaluate the soil Cu status in citrus orchards and improve citrus fruit quality by regulating and controlling soil Cu levels and citrus tree Cu status.

## 2. Results

### 2.1. Characteristics of Cu Concentrations in Soil, Leaf, and Fruit

**Figure 1:**
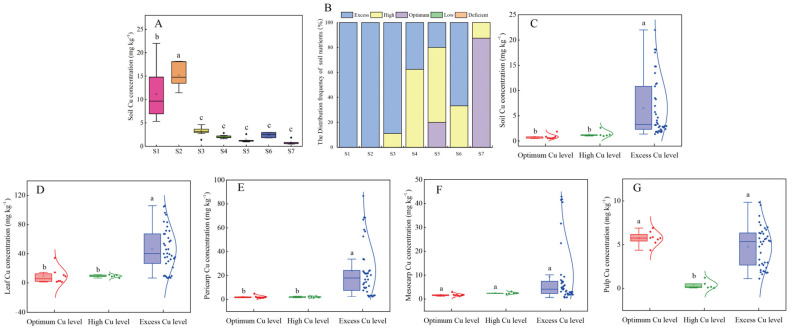
The Cu concentrations of soil, leaf, and fruit. (**A**) Soil available Cu concentrations in different orchards; (**B**) the classification of soil available Cu in different orchards; the Cu concentrations of soil available Cu (**C**), leaf Cu (**D**); pericarp Cu (**E**); mesocarp Cu (**F**); pulp Cu (**G**) under different soil Cu levels. The different letters above the box plots indicate significant differences between different soil Cu levels at 5% level (*p* < 0.05; n_s1_ = 10, n_s2_ = 5, n_s3_ = 9, n_s4_ = 8, n_s5_ = 5, n_s6_ = 6, n_s7_ = 8; n_opimum Cu level_ = 8, n_high Cu level_ = 5, n_excess Cu level_ = 38).

The soil available Cu concentrations of seven citrus orchards ranged from 0.5 to 22.0 mg kg^−1^, with an average of 5.1 mg kg^−1^ and a variation coefficient of 108.9% (Figure 1A). The soil available Cu gradings of citrus orchards were classified as follows: the proportion of excessive grading samples reached up to 100% in both S1 and S2 orchards, 88.9% in the S3 orchard, and 66.7% in the S6 orchard; the proportion of high grading samples reached up to 62.5% in the S4 orchard and 60.0% in the S5 orchard; and the proportion of optimum grading samples reached up to 87.5% in the S7 orchard (Figure 1B). Five soil Cu levels of deficient (<0.3 mg kg^−1^), low (0.3–0.5 mg kg^−1^), optimum (0.5–1.0 mg kg^−1^), high (1.0–2.0 mg kg^−1^), and excess (>2.0 mg kg^−1^) were sorted according to soil available Cu concentration. Thus, seven orchards were sorted into three soil Cu levels with the average soil available Cu concentration of each orchard: excess Cu level (S1–S4, S6), high Cu level (S5), and optimum Cu level (S7). The soil available Cu threshold concentrations of excess Cu level, high Cu level, and optimum Cu level were 6.5, 1.4, and 0.8 mg kg^−1^, respectively (Figure 1C). These results indicate that orchards with excess Cu level reached up to 71.4%, while there was no Cu deficient orchard.

Leaf Cu concentrations of seven citrus orchards ranged from 1.3 to 105.9 mg kg^−1^, with an average of 37.1 mg kg^−1^ and a variation coefficient of 81.8%, which varied greatly among different orchards (Figure S2A). The classification of leaf Cu concentration grading of different orchards was as follows: the proportion of excessive grading samples reached up to 100%, including S1–S3 and S6 orchards, the proportion of high grading samples reached up to 100%, including S4 and S5 orchards, the proportion of deficient grading samples reached up to 50.0%, including S7 orchard (Figure S2B). It could be seen in Figure 1D that the leaf Cu concentration (46.5 mg kg^−1^) at an excess Cu level was significantly higher than those (9.4 and 9.6 mg kg^−1^) at high Cu level and optimum Cu level, respectively. To conclude, the results suggest that leaf Cu concentrations in orchards were mainly in the excess state, and soil Cu in excess levels can significantly increase Cu accumulation in the leaf.

Fruit Cu concentrations in seven citrus orchards varied greatly (Figure S2C). The Cu concentration of fruit pericarp, mesocarp, and pulp ranged 1.4–86.8 mg kg^−1^, 0.6–42.9 mg kg^−1^, and 0.1–10.2 mg kg^−1^, with an average of 18.1, 7.8, and 5.4 mg kg^−1^, and the variation coefficients were 144.1, 118.1, and 47.1%, respectively. Moreover, pericarp Cu concentration at excess Cu level (23.7 mg kg^−1^) was higher than those at high Cu level (2.0 mg kg^−1^) and optimum Cu level (2.0 mg kg^−1^), respectively (Figure 1E). In addition, pulp Cu concentration was highest (6.0 mg kg^−1^) at excess Cu level and lowest (0.4 mg kg^−1^) at high Cu level (Figure 1G). Taken together, among the three Cu levels, our results suggest that Cu concentration of fruit follows pericarp > mesocarp > pulp. Soil Cu in excess levels significantly increased the pericarp Cu concentration.

Overall, these results demonstrate that soil Cu and leaf Cu in seven orchards are primarily at the excess level. Soil Cu in excess levels highly accumulates in the leaf and frui.

### 2.2. Characteristics of Citrus Fruit Quality

As shown in Figure S3, fruit weight, pericarp thickness, pericarp color value, juice yield, TSS, TA, TSS/TA, and Vc in seven orchards ranged 14.3–192.8 g, 1.4–3.7 cm, 4.5–32.9, 47.7–68.0%, 8.3–15.0 °Brix, 0.1–1.0%, 10.1–161.8, and 11.7–27.6 mg 100 g^−1^, with the average of 94.5 g, 2.4 cm, 23.8, 58.0%, 12.8 °Brix, 0.40%, 43.4, and 19.1 mg 100 g^−1^, and a variation coefficient of 43.1, 26.7, 35.8, 7.8, 12.0, 46.7, 69.2, and 20.8%, respectively.

**Figure 2:**
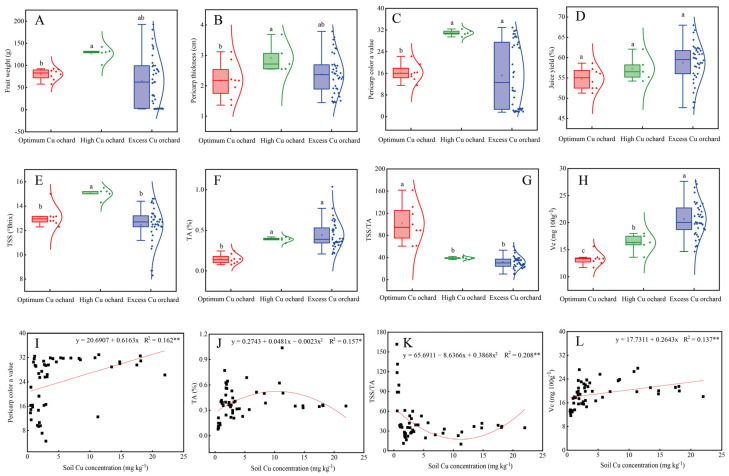
The characteristics of fruit qualities under different soil Cu levels (**A**–**H**) and the regression analysis of pericarp color a value (**I**), TA (**J**), TSS/TA (**K**), and Vc (**L**) with the increasing soil available Cu concentrations. The different letters above the box plots indicate significant differences between different soil Cu levels at 5% level (*p <* 0.05; n_opimum Cu level_ = 8, n_high Cu level_ = 5, n_excess Cu level_ = 38). * and ** superscripts after R^2^ values indicate statistical differences at *p* < 0.05 and *p* < 0.01, respectively.

Figure 2A–H show the characteristics of fruit quality under different soil Cu levels. In detail, pericarp color value, TSS, TA, TSS/TA, and Vc were significantly affected by soil Cu levels (Figure 2A–H). Interestingly, pericarp color a value, TA, and Vc were significantly higher with 90.0, 166.6, and 22.4% in high Cu levels and 50.5, 204.2, and 55.3% in excess Cu levels, while TSS/TA was significantly lower with 68.7% in high Cu levels and with 61.6% in excess Cu levels. According to PCA, we found that the comprehensive scores of different soil Cu levels was ordered as follows: high Cu level (70.9) > excess Cu level (59.2) > optimum Cu level (50.1), indicating that high Cu level could achieve better fruit quality supported by higher pericarp color a value, TSS, TA, and Vc compared with optimum Cu level (Tables S2 and S3). In detail, with the increase of soil Cu levels, pericarp color a value and Vc sharply improved (Figure 2I,J). Surprisingly, there was a dose effect of soil Cu concentration on TA with a trend of rising first then falling, and TSS/TA with a trend of falling first then rising (Figure 2K,L). Taken together, TA was the most sensitive response factor to soil Cu levels. Pericarp color, sugar, and acid in citrus fruit were affected by soil Cu levels.

### 2.3. Relationships of Soil Cu, Leaf Cu, and Fruit Cu

**Figure 3:**
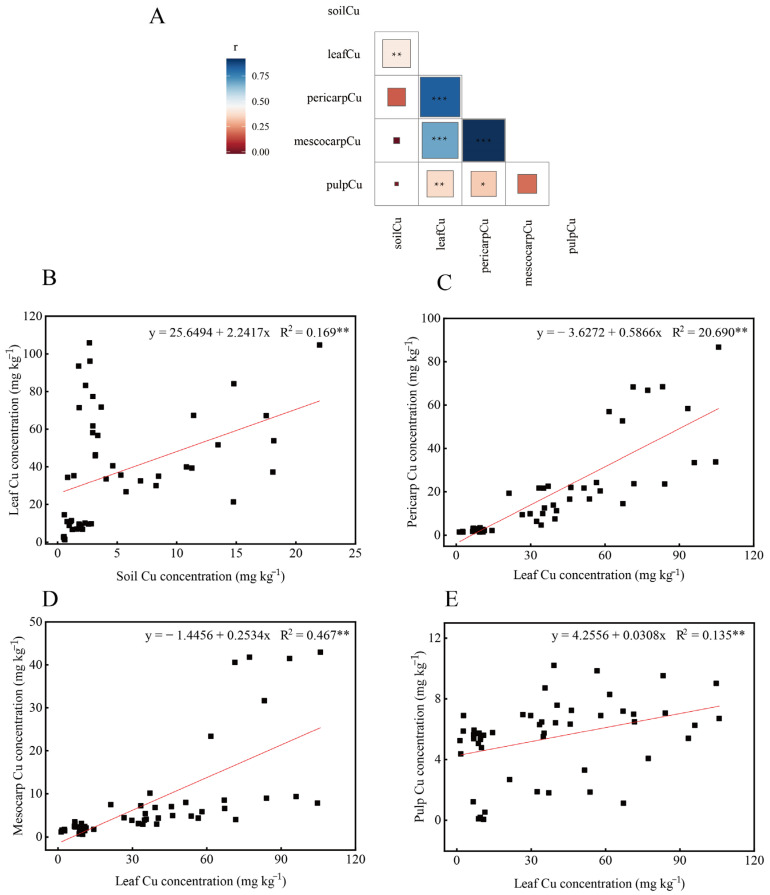
Correlation analysis and linear regression analysis of soil available Cu versus plant Cu in orchards under different soil Cu levels. (**A**) Correlation analysis; (**B**–**E**) linear regression analysis. *, ** and *** represented in the heatmap and superscripted after R^2^ values indicate statistical differences at *p* < 0.05, *p* < 0.01, *p* < 0.001, respectively.

As shown in Figure 3A, soil Cu showed a significant positive relationship with leaf Cu. Likewise, leaf Cu was strongly positively correlated with pericarp Cu, mesocarp Cu, and pulp Cu. Thus, leaf Cu rose as soil Cu increased (Figure 3B), and a rise in leaf Cu promoted fruit Cu accumulation (Figure 3C–E). To sum up, we conclude that the rise in soil Cu promotes leaf Cu accumulation, and the rise in leaf Cu leads to a rise in fruit Cu.

### 2.4. Relationships between Fruit Quality and Soil-Tree Cu

**Figure 4:**
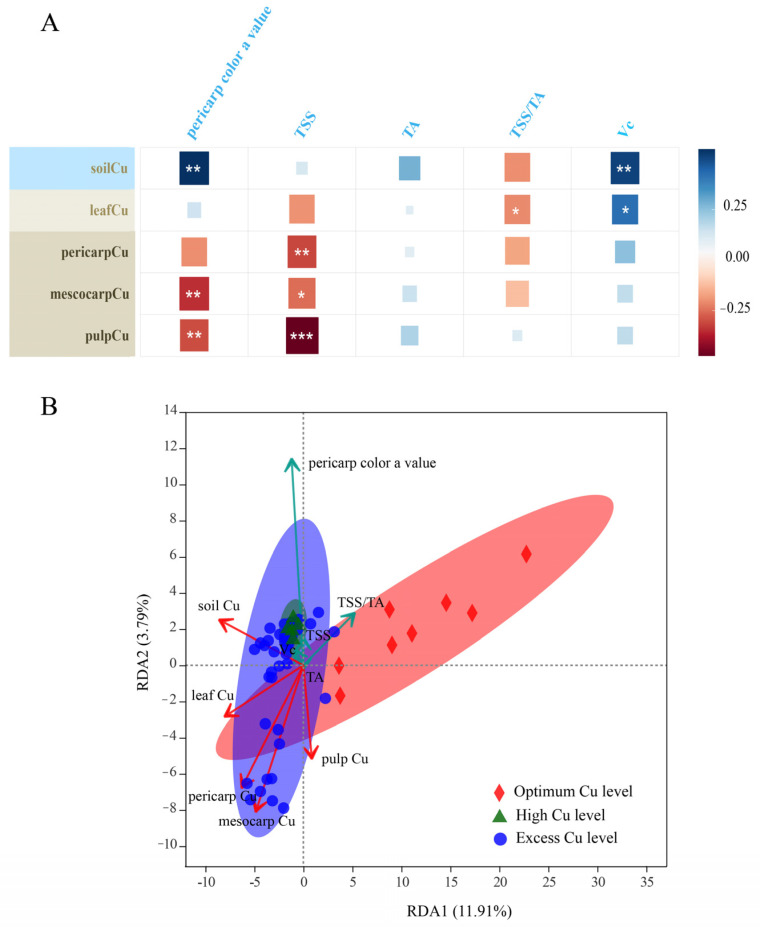
Correlation analysis (**A**) and redundancy analysis of soil-plant Cu versus fruit quality (**B**). * presented for *p* < 0.05, ** presented for *p* < 0.01, *** presented for *p* < 0.01, respectively.

From Figure 4A, the color parameter a* value was significantly positively correlated with soil Cu but negatively correlated with fruit Cu (pericarp Cu, mesocarp Cu, and pulp Cu). Likewise, there was a significantly negative relationship between TSS and fruit Cu, and between TSS/TA and leaf Cu. However, Vc was significant positively correlated with soil Cu and leaf Cu. 

**Figure 5:**
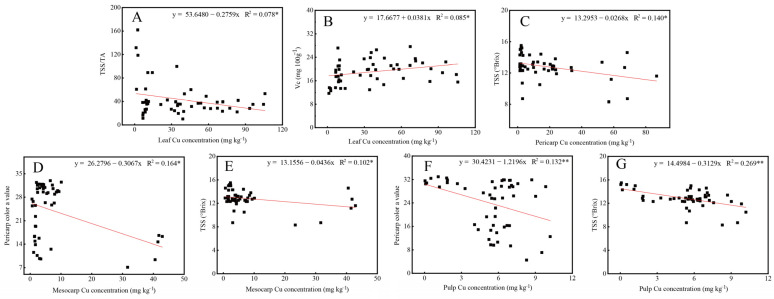
Linear regression analysis of soil-plant Cu concentrations versus different fruit qualities of citrus (n = 51). (**A**) leaf Cu versus TSS/TA, (**B**) leaf Cu versus Vc, (**C**) pericarp Cu versus TSS, (**D**) mesocarp Cu versus pericarp color a value, (**E**) mesocarp Cu versus TSS, (**F**) pulp Cu versus pericarp color a value, (**G**) pulp Cu versus TSS. * and ** superscripts after R^2^ values indicate statistical differences at *p* < 0.05 and *p* < 0.01, respectively.

The data in Figure 4B revealed that orchards of excess Cu level and high Cu level clustered together, and separated with orchards of optimum Cu level. These results suggested that fruit qualities of excess Cu level and high Cu level contained many similarities, and had a significant difference from those in optimum Cu level. Furthermore, the effects of soil-tree Cu on fruit quality were as follows: soil Cu > fruit Cu (mesocarp Cu > pericarp Cu > pulp Cu) > leaf Cu (Table S4), indicating that soil Cu was the main factor that affected fruit quality. Moreover, Figure 4B also highlights that pericarp color a value was positively correlated with soil Cu, but it was negatively with fruit Cu. TSS was negatively correlated with fruit Cu, TSS/TA was negatively correlated with leaf Cu, while Vc was positively correlated with soil Cu and leaf Cu. These results were consistent with those in Figure 4A. As a result, as leaf Cu increased, TSS/TA decreased but Vc increased (Figure 5A,B). In addition, TSS is reduced with the increase of pericarp Cu, mesocarp Cu, and pulp Cu. Likewise, pericarp color a value was reduced with the increasing mesocarp Cu and pulp Cu (Figure 5C–G). 

**Figure 6:**
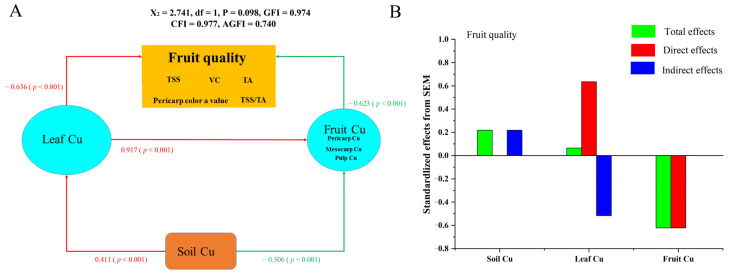
The direct and indirect effects of fruit quality with soil Cu, leaf Cu, and fruit Cu was determined by structural equation modeling (SEM). (**A**) The structural equation modeling diagram. (**B**) The direct and indirect effects of different factors on fruit quality. Positive and negative effects were represented by red and green colors, respectively. The red line indicated a significant positive correlation, the green line indicated a significant negative correlation. *p* < 0.05 and *p* < 0.001 denote significant and extreme difference, respectively.

Importantly, structural equation modeling (SEM) was used to systematically analyze how soil-tree Cu affects fruit quality in citrus (Figure 6). We found that fruit Cu had a direct adverse effect on citrus quality; an increase in fruit Cu led to a decrease in TSS and pericarp color value (Figure 5C–G). In contrast, leaf Cu significantly improved citrus fruit quality with direct and indirect effects. This result showed that an increase in leaf Cu gave rise to Vc, as shown in Figure 5B. Nevertheless, soil Cu affected fruit quality indirectly by improving leaf Cu and reducing fruit Cu. 

In conclusion, fruit quality in excess Cu orchards and high Cu orchards contained many similarities and had a significant difference from those in optimum Cu orchards. Soil Cu is the main factor affecting fruit quality. Fruit Cu reduced fruit quality supported by the decrease of TSS and pericarp color a value by direct effects. Leaf Cu improved fruit quality supported by the increase of Vc with direct and indirect effects. Soil Cu strongly affects pericarp color a value, TSS, TA, TSS/TA, and Vc by regulating fruit Cu and leaf Cu. 

## 3. Discussion

### 3.1. The Effects of Soil-Tree Cu on Sugars Contents in Citrus

It has been reported that Cu is accumulating in citrus orchard soil year after year due to the application of Cu-containing fungicides [9,12] and organic fertilizers [35,36]. It is well known that Cu is a component of a variety of enzymes, and is involved in photosynthesis, respiratory, carbohydrate metabolism, as well as oxidation-reduction reactions [37], suggesting that Cu plays an important role in the formation of fruit quality. Our results showed that TSS was significantly negatively correlated with fruit Cu, and decreased with the increase of fruit Cu by its direct effects (Figure 4A, Figure 5C,E,G, and Figure 6), indicating that fruit sugar was affected by Cu. In the case of total sugars in citrus, sucrose exhibits the highest content, and it is determined by sucrose metabolism enzymes [38,39]. Sucrose synthase (SS) is an extremely important enzyme in sucrose metabolism, which regulates the resynthesis and degradation of sucrose [40]. A previous study reported that Cu was significantly negatively correlated with sucrose in the ‘Chang fu 2′ apple [41]. Lothar (1995) [42] observed that SS activity can be suppressed by Cu^2+^, leading to an inhibition of sucrose synthesis. Hence, the reason why citrus fruit Cu decreased sugars may be related to the reduction of sucrose due to the weaker synthase activity suppressed by Cu^2+^. Meanwhile, a large amount of Cu in plants will cause oxidative stress and produce harmful reactive oxygen species (ROS) [34], which disrupt the balance of the redox system, change the primary carbon metabolism pathway, finally improving fruit sugar decomposition [43,44]. As a result, the reduction of sugar by Cu in citrus fruit also may attribute to sugar decomposition.

In citrus fruit, approximately 50% of sucrose in the fruit is transported from leaf photosynthesis during fruit development [45], indicating that sugar accumulation in fruit is also affected by leaf photosynthesis. In our work, leaf Cu was negatively correlated with the TSS and TSS/TA ratio, which was consistent with previous findings [46]. Previously, it was found that when there is too much Cu in the leaf, chlorophyll biosynthesis is inhibited, pigment content and composition are reduced, leaf gas exchange is disrupted, photosystem II is destroyed, and finally photosynthesis declines [47,48]. To conclude, in our work, high Cu concentrations in leaves will inhibit photosynthetic product accumulation, resulting in a decrease in fruit sugar.

### 3.2. The Effects of Soil-Tree Cu on Acids in Citrus

Organic acids in citrus are responsible for sourness and up to 90% of them are citric acid [49,50], which plays a crucial role in fruit flavor, quality, and the maturity of most types of fruits [39]. A previous study reported that soil available Cu was significantly positively correlated with TA, while negatively correlated with the ratio of TSS/TA under soil Cu concentrations ranging 0.44~2.24 mg kg^−1^ in the citrus orchards cultivating “tian orange”, “jin orange”, and “Xia orange” all grafted on trifoliate orange [*Poncirus trifoliata* (L.) Raf] [51]. Likewise, the TA of Orah mandarin [52] and “Nanfeng” tangerine [53] grafted on trifoliate orange [*Poncirus trifoliata* (L.) Raf.] was positively correlated with soil available Cu under soil Cu concentrations ranging 0.15–2.48 mg kg^−1^ and 0.35–37.23 mg kg^−1^, respectively. This study was in accordance with these results, indicating that there was a consistent effect of soil Cu on acid content in citrus fruits of different varieties. Noteworthy, citrus fruit quality is also influenced by cultivar [54] and rootstock [55]. Hence, citrus fruit characteristics with the same cultivar and rootstock under different soil Cu levels should be further studied in the future. Conversely, when soil available Cu concentration ranged from 3.32 to 92.72 mg kg^−1^, the TA of “Jinsha” pomelo was negatively correlated with soil available Cu [56]. These results indicate that the relationship between TA with soil Cu may be related to the Cu concentrations in the soil. In our study, there was a dose effect of soil Cu concentration on TA with a trend of rising first (soil available Cu concentration ranged from 0.5–10.7 mg kg^−1^) and then falling (soil available Cu concentration ranged from 10.7–22.0 mg kg^−1^) (Figure 2J), suggesting that low soil available Cu increased TA, while high soil available Cu decreased TA. Although TA was increased with the increase of soil Cu, TA had no significant difference with leaf Cu and fruit Cu, indicating that fruit TA was not affected directly by leaf Cu and fruit Cu or they had a more complicated relationship. As shown in a previous study, Cu has a high affinity to organic acids [57]. Hence, as an organic chelator, citric acid facilitates Cu transport in the phloem [58]. These results imply that fruit TA may be involved in Cu transportation. 

At present, there is little information about the relationship of fruit TA and Cu, so it is necessary to investigate more interactions between Cu and acids. The main organic acid in citrus fruits is citric acid (CA), which accounts for 70–90% of organic acid [59]. These three processes determine CA accumulation: synthesis takes place in mitochondria worked with citrate synthetase (CS) and phosphoenolpyruvate carboxylase (PEPC), while decomposition occurs in the cytoplasm performed by aconitase (ACO) and isocitrate dehydrogenase (IDH), finally, CA is primarily stored in vacuoles [60,61,62,63]. 

### 3.3. The Effects of Soil-Tree Cu on Pericarp Color in Citrus

Yellow, orange, and red colors in citrus fruits are caused by the accumulation of carotenoids [64], which determine fruit appearance quality and play a vital role in nutritional value and health care function [65,66]. The previous study showed that the synthesis of carotenoids was affected by light, hormones, temperature, nutrient supply, etc. [67,68]. In this work, the pericarp color value was significantly negatively correlated with fruit Cu (Figure 4A), indicating that the change of the pericarp color value (represented red and green) under different soil Cu levels were possibly associated with carotenoid synthesis. Lycopene is one of the main pigments in citrus fruit and is regarded as an important intermediate substance in carotenoid synthesis, giving red color to fruits [65]. However, lycopene was unstable to many metal ions, especially metal ions with strong oxidation abilities such as Cu^2+^, which can cause oxidative damage by high Cu^2+^ concentrations [69]. It has been reported that lycopene significantly decreased from 54.91 mg 100 g^−1^ (DW) to 38.21 mg 100 g^−1^ (DW) with the increase of Cu NPs (0, 10, 50, 250 mg L^−1^) in tomato fruit [70]. Therefore, in our work, the pericarp color a value decreased (getting greener) as the increase of fruit Cu may be due to the reduction of lycopene, which was oxidated by Cu^2+^ leading to an inhibition of carotenoid synthesis. However, the pericarp’s greenish color will decrease citrus fruit quality; more attention should be paid to regulating and controlling fruit Cu concentration. 

Moreover, sugar is not only the basic substance of carotenoid biosynthesis but also regulates the process of chloroplast to chromosomes during fruit ripening and senescence [71]. Previous studies reported that carotenoid in the skin was significantly positively related to glucose, fructose, and sucrose in the pulp of figs (*Ficus carica* L.) [72]. Huff (1984) [73] and Iglesis et al. (2001) [74] found that late-ripening sweet oranges return to green again when sugar concentration drops in the peel in spring. During the fruit ripening period, spraying sucrose, fructose, and glucose on the crown of the tree can improve the peel color of the pear [75]. In our study, there was a significant positive correlation between TSS and pericarp color value (Figure S4), suggesting that the greenish pericarp might be caused by the lack of sugar, which prevents carotenoid biosynthesis. 

## 4. Materials and Methods

### 4.1. Citrus Orchards

The samples of soil, leaf, and fruit were collected in December 2019, from seven representative citrus orchards in Wuming district (S1 and S2), Shanglin district (S3), Lingui district (S4), Linchuan district (S5), Mengshan district (S6), and Bobai district (S7), in Guangxi province, China (Figure S1). This area has a subtropical monsoon climate with average temperature, precipitation, and annual sunshine hours of 21.6 °C, 1335.3 mm, and 1675.1 h, respectively [76]. The average soil pH and organic matter of each orchard ranged 3.84–6.13 and 16.4–43.25 g kg^−^^1^, respectively; 85.7% orchards soil were at acid level (pH < 5.4) and rich level (>30 g kg^−^^1^). The citrus varieties were Orah mandarin grafted on Citrus junos Tanaka, or C. *reticulata Blanco*, or *Poncirus trifoliata* (L.) Raf, respectively, Gonggan mandarin grafted on *Poncirus trifoliata* (L.) Raf, and Mashuiju tangerine grafted on C. *sunki Hort* (Table S1). There were five to ten uniformly grown trees of four to seven-year-old that were randomly selected according to the “S” shaped line sampling points from each orchard. 

### 4.2. Sampling

Soil sampling: two soil sampling points were taken diagonally from each tree avoiding the roadside, fertilization point, and drip irrigation moist area. Soil samples were collected within 10 cm of the citrus tree canopy drip line. After removing the surface vegetation and organic cover, 0–30 cm depth soil was collected, and about 500 g of composite soil samples were taken out by the quartering method and put into a plastic bag [77]. The collected soil samples were quickly taken back to the laboratory for impurity removal, air drying, grinding, screening, and storage.

Leaf and fruit sampling: the fruits of the current year’s spring shoots were collected in the middle of the outer canopy in four directions east, south, west, and north. In addition to the 2nd and 3rd complete disease-free leaves (including petioles) of the spring shoots, fruits of uniform size and ripeness were collected under the prevailing weather conditions. The collected leaves and fruits were quickly taken back to the laboratory and washed with 0.1% neutral detergent, clean water, 0.2% HCl, and deionized water in sequence within 2 min. The samples of pericarp, mesocarp, and pulp of fruit were separated after the leaves and fruits were sucked dry, which were deactivated at 105 °C for 30 min. They were then dried at 65 °C until constant weight and pulverized into a powder which were then stored in a bag under dry conditions. Additionally, approximately 80 g of fresh pulp was separated in order to measure the fruit quality.

### 4.3. Analysis Method

Soil pH was determined in a suspension (soil:water = 1:2.5, *w*/*v*) in deionized water with a digital pH meter (FE20/EL20, Shanghai Mettler Toledo Co., Shanghai, China). The concentrations of soil organic matter (SOM) were measured by the potassium dichromate volumetric method [77]. Soil available Cu was obtained by extracting 10 g of dry soil (sieved to 2 mm) with 20 mL of diethylenetriamine pentaacetic acid (DTPA) solution (0.005 mol L^−^^1^ DTPA + 0.01 mol L^−^^1^ CaCl_2_ + 0.1 mol L^−^^1^ triethanolamine, pH 7.3) [78]. The concentrations of Cu in leaves and fruits were measured by mixed acid (HNO_3_:HClO_4_ = 4:1, *v/v*) digestion and determined by atomic absorption spectrometer (Z-2000, HITACHI, Tokyo, Japan) [79]. Fruit weight (g) was measured by the weighing method. Peel thickness (cm) was measured by vernier calipers. The juice yield is the percentage of the weight of squeezed fruit juice in the total weight of the fruit. Where juice yield (%) = (juice weight/pulp weight) × 100%. Total soluble solid (TSS) was measured using a handheld digital sugar meter (ATAGO PAL-1, Tokyo, Japan). Titratable acid (TA) was determined by neutralization titration with 0.05 mol L^−^^1^ NaOH [80]. Vitamin C (Vc) was determined by 2,6-dichlorophenolindiophenol titration [81].

### 4.4. Evaluation Standard

Cu nutrient grading standards were based on relevant studies [82,83,84]. Cu levels in soil were divided into deficient (<0.3 mg kg^−^^1^), low (0.3–0.5 mg kg^−^^1^), optimum (0.5–1.0 mg kg^−^^1^), high (1.0–2.0 mg kg^−^^1^), and excess (>2.0 mg kg^−^^1^). Cu levels in leaves were divided into deficient (<3 mg kg^−^^1^), low (3–5 mg kg^−^^1^), optimum (5–16 mg kg^−^^1^), high (16–20 mg kg^−^^1^), and excess (>20 mg kg^−^^1^).

### 4.5. Statistical Analysis

Data statistical analyses were conducted using one-way analysis of variance (ANOVA) and significant differences among the means were determined by the Duncan test at *p* < 0.05 using the IBM SPSS Statistics 20.0 analytical software. The results were described as means ± standard deviation (SD). R software (RStudio Inc., Seattle, DC, USA) (version 3.3.1) with packages “linkET”, “ggplot2”, “dplyr”, “Hmisc”, were used to operate correlation analysis; packages “ambient” and “ggtext” were used to carry out regression analysis; packages “rfPermute” and “ggtext” were used to perform random forest analysis; package “lavaan” was used to conduct redundancy analysis (RDA). Histogram figures were drawn by the Origin 9.0 (OriginLab Inc., Northampton, MA, USA) software. Principal component analysis (PCA) was conducted by SPSS 20.0 (SPSS Inc., Chicago, IL, USA).

## 5. Conclusions

Collectively, pericarp color a value, TSS, TA, TSS/TA, and Vc were strongly affected by soil Cu levels. Especially, pericarp color a value, TA, and Vc were higher, while TSS/TA was lower in high and excess Cu levels compared to optimum Cu level. The increase of fruit Cu reduced fruit quality as mainly supported by greener pericarp and the decrease of TSS by direct effects. The increase of leaf Cu increased fruit quality as primarily assessed by the increase of Vc by performing with direct and indirect effects. Moreover, the increased of soil Cu strongly affected fruit quality, as supported by the increase of pericarp color a value and Vc. TA had a trend of rising first then falling, and TSS/TA had a trend of falling first then rising by indirect effects via up-regulating leaf Cu or down-regulating fruit Cu. Therefore, reasonable regulation and control of the application of Cu-containing fungicides and fertilizers to maintain soil Cu at high levels can effectively increase pericarp color together with sugar and acid accumulation in citrus fruit.

## Supplementary Materials

The following supporting information can be downloaded at: https://www.mdpi.com/article/10.3390/plants11212943/s1, Figure S1: Distribution of sampling sites; Figure S2: The Cu concentrations of leaf and fruit in different orchards; Figure S3: Characteristics of fruit quality in different orchards; Figure S4: Correlation analysis of different fruit qualities in citrus; Table S1: Basic information of citrus orchards; Table S2: Principal component analysis of fruit quality indicators in different orchards; Table S3: Fruit quality scores in orchards with different soil Cu levels; Table S4: The datasheet for environmental factors in RDA of fruit quality with different Cu levels.
