# Supplementary material for: Fruit Characteristics of Citrus Trees Grown under Different Soil Cu Levels
Source: Plants (Basel). 2022 Nov 1;11(21):2943. doi: 10.3390/plants11212943 (PMC9657546; doi:10.3390/plants11212943)
Supplement: Supplementary file 1 [file plants-11-02943-s001.zip › plants-1968170-supplementary.pdf]

Supplementary Figure

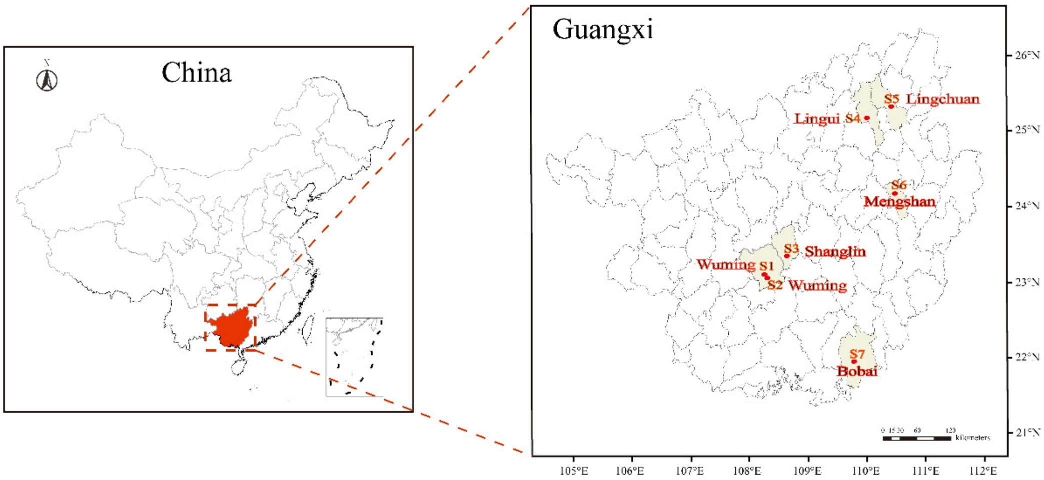

Figure S1. Distribution of sampling sites.

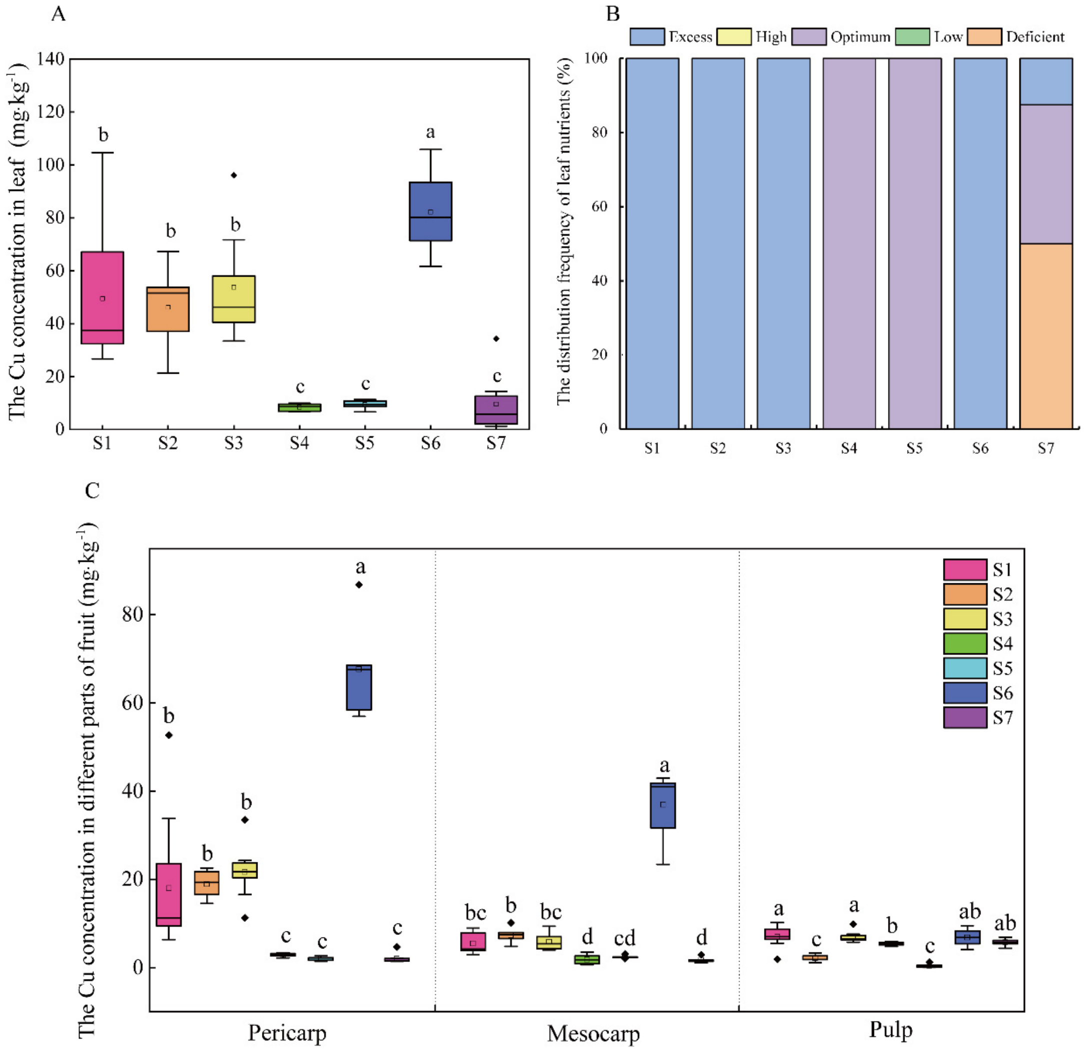

Figure S2. The Cu concentrations of leaf and fruit in different orchards. A: leaf Cu concentrations; B: the classification of leaf Cu in different orchards; C: fruit concentrations. The different letters indicated significant differences between sites at 5% level ($P < 0.05$; $n_{s1}=10$, $n_{s2}=5$, $n_{s3}=9$, $n_{s4}=8$, $n_{s5}=5$, $n_{s6}=6$, $n_{s7}=8$).

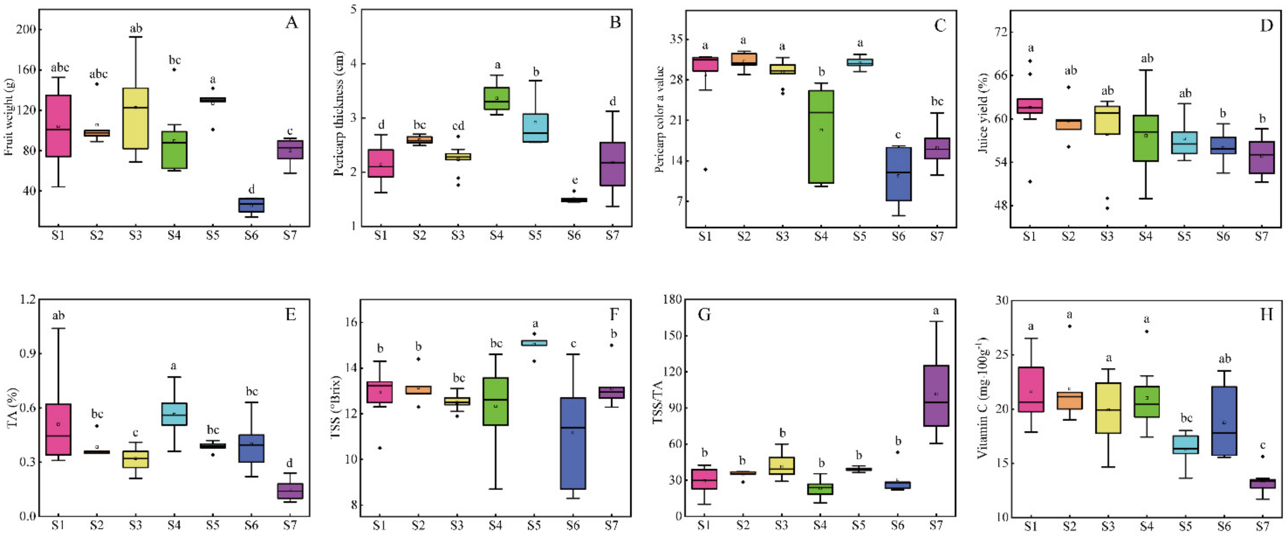

Figure S3. Characteristics of fruit quality in different orchards. The different letters indicated significant differences between sites at 5% level ($P < 0.05$; n $_{s1}$=10, n $_{s2}$=5, n $_{s3}$=9, n $_{s4}$=8, n $_{s5}$=5, n $_{s6}$=6, n $_{s7}$=8).

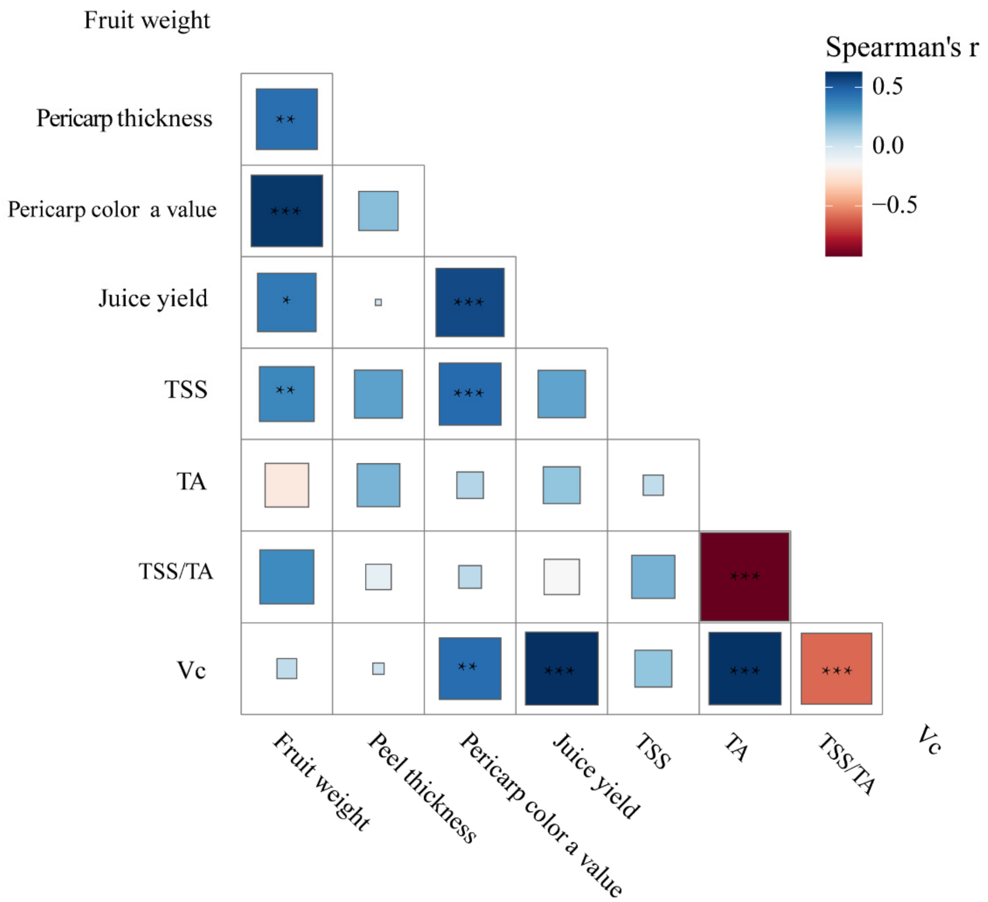

Figure S4. Correlation analysis of different fruit qualities in citrus. *, ** and *** represented in the heatmap indicate statistical differences at $P < 0.05$, $P < 0.01$, $P < 0.001$ respectively.

Table S1

Basic information of citrus orchards.

Samples area				Sample/ trees	Variety	Stock	Age/a
Orchard	Site	Longitude	Latitude				
S1	Shuangqiao Town in Wuming District of Guangxi	108°18'13"	23°5'39"	10	Orah mandarin	*Citrus junos* Tanaka	4
S2	Shuangqiao Town in Wuming District of Guangxi	108°17'22"	23°6'19"	5	Orah mandarin	*C.reticulata* Blanco	7
S3	Mingliang Town in Shanglin District of Guangxi	108°37′48″	23°24′5″	9	Orah mandarin	*Poncirus trifoliata* (L.) Raf	6
S4	Liangjiang Town in Lingui District of Guangxi	110°9′53″	25°17′6″	8	Orah mandarin	*Poncirus trifoliata* (L.) Raf	4
S5	Lingtian Town in Lingchuan District of Guangxi	110°27'35"	25°20'49"	5	Orah mandarin	*Poncirus trifoliata* (L.) Raf	5
S6	Hexi Town in Mengshan District of Guangxi	110°35'32"	24°11'4"	6	Gonggan mandarin	*Poncirus trifoliata* (L.) Raf	6
S7	Dongping Town in Bobai District of Guangxi	109°49'27"	22°3'19"	8	Mashuiju tangerine	*C.sunki* Hort	7

Table S2

Principal component analysis of fruit quality indicators in different orchards.

Principal component	Eigenvalue	Variance contribution /%	Accumulative variance contribution /%
1	2.893	36.166	36.166
2	2.295	28.690	64.858
3	1.204	15.048	79.904

Table S3

Fruit quality scores in orchards with different soil Cu levels.

Soil Cu levels	Score	Ranking
Optimum Cu level	54.1	3
High Cu level	70.9	1
Excess Cu level	59.2	2

Table S4

The datasheet for environmental factors in redundancy analysis (RDA) of fruit quality with different Cu levels.

	RDA1	RDA2	r^2	*p*-values
Soil Cu	-0.589	0.808	0.199	**0.007**
Leaf Cu	-0.946	-0.323	0.079	0.147
Pericarp Cu	-0.047	-0.999	0.171	**0.020**
Mescocarp Cu	0.088	-0.996	0.250	**0.002**
Pulp Cu	0.300	-0.954	0.137	**0.035**
